# Supplementary material for: Confounding effects of heart rate, breathing rate, and frontal fNIRS on interoception
Source: Sci Rep. 2022 Nov 30;12:20701. doi: 10.1038/s41598-022-25119-z (PMC9712694; doi:10.1038/s41598-022-25119-z)
Supplement: Supplementary file 1 — Supplementary Figures. [file 41598_2022_25119_MOESM1_ESM.docx]

SUPPLEMENTARY FIGURES

# Confounding effects of heart rate, breathing rate, and frontal fNIRS on interoception

Diego Candia-Rivera ^1*^, M. Sofía Sappia ^2, 3^, Jörn M. Horschig ^2^, Willy N. J. M. Colier ^2^, Gaetano Valenza ^1^

^1^ Bioengineering and Robotics Research Center E. Piaggio & Department of Information Engineering, School of Engineering, University of Pisa, 56122, Pisa, Italy

^2^ Artinis Medical Systems, B.V., Einsteinweg 17, 6662 PW, Elst, The Netherlands

^3^ Radboud University Nijmegen, Donders Institute for Brain, Behaviour and Cognition, 6525 EN, Nijmegen, The Netherlands

* Corresponding author: [diego.candia.r@ug.uchile.cl](mailto:diego.candia.r@ug.uchile.cl)


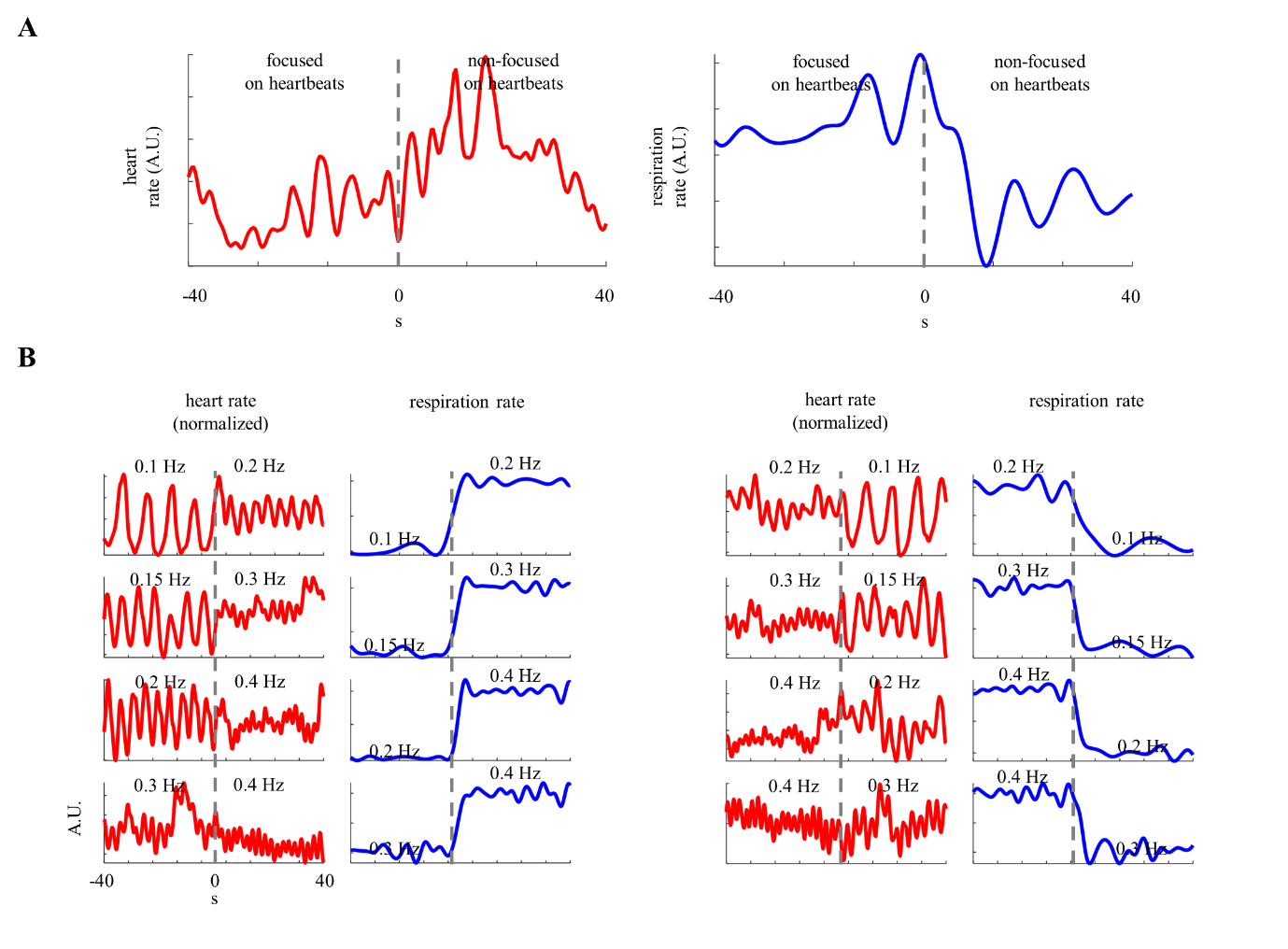


Supplementary Figure 1. Induced respiratory modulations on heart rate in a single subject. (Panel 1 A): Relative change in heart and breathing rates triggered by the transition from the performance to the cessation of focusing on heartbeats. Heart rate series were smoothed using a 10-second moving average window and a one-sample step. Data corresponds to an average over six trials. (Panel B) Relative change in heart and breathing rates expressed in Hz during slower-to-faster and faster-to-slower breathing rate transitions. All signals are z-score normalized over a window of -40–40 seconds with respect to each transition in breathing rate.


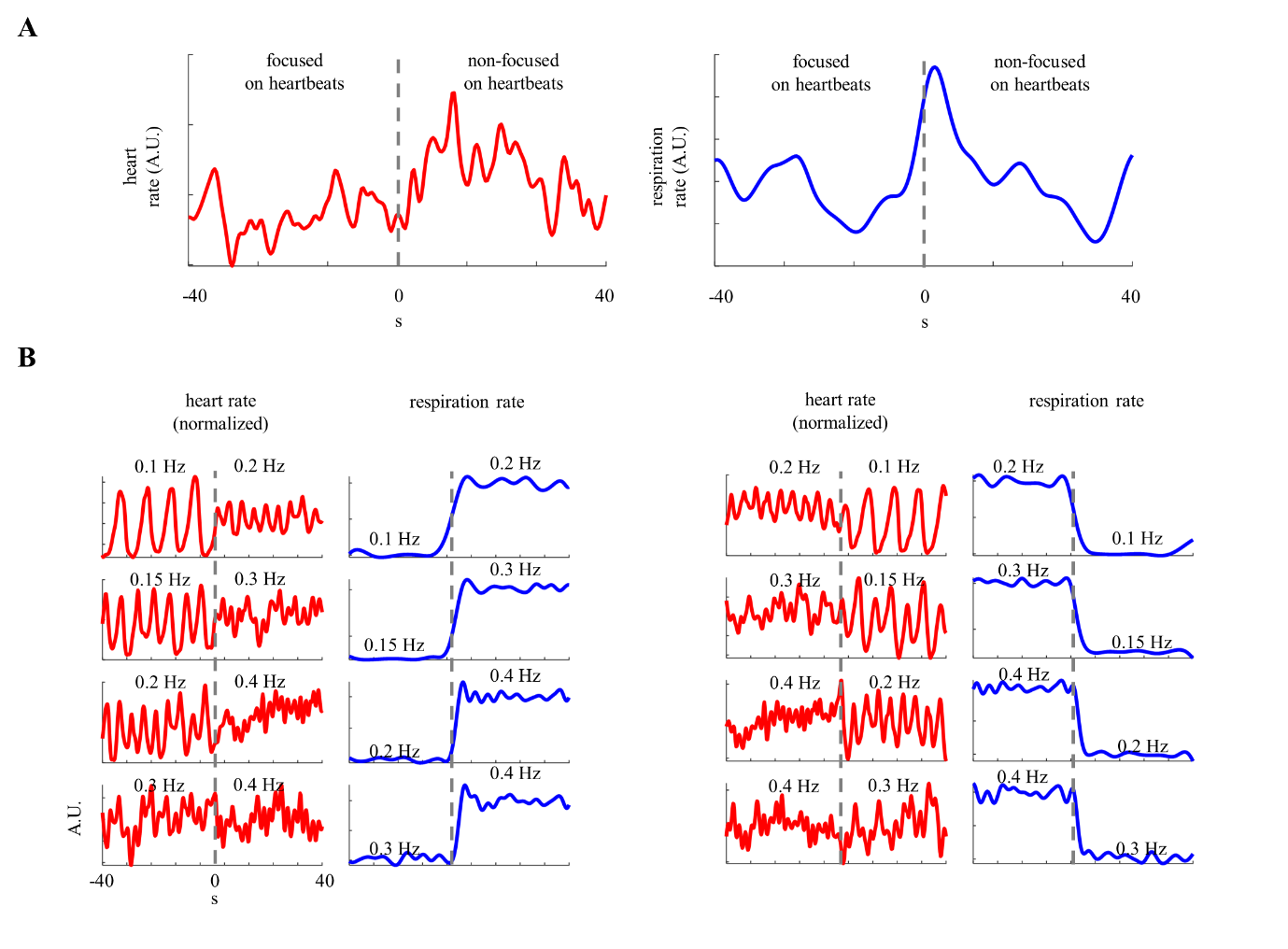


Supplementary Figure 2. Induced respiratory modulations on heart rate in a single subject. (Panel 1 A): Relative change in heart and breathing rates triggered by the transition from the performance to the cessation of focusing on heartbeats. Heart rate series were smoothed using a 10-second moving average window and a one-sample step. Data corresponds to an average over six trials. (Panel B) Relative change in heart and breathing rates expressed in Hz during slower-to-faster and faster-to-slower breathing rate transitions. All signals are z-score normalized over a window of -40–40 seconds with respect to each transition in breathing rate.
